# Is night‐time surgical procedure for renal graft at higher risk than during the day? A single center study cohort of 179 patients

**DOI:** 10.1002/iid3.566

**Published:** 2021-11-18

**Authors:** Patrick Julien Treacy, Flora Barthe, Imad Bentellis, Ugo Giovanni Falagario, Thomas Prudhomme, Laetitia Imbert de La Phalecque, Aysha Shaikh, Laetitia Albano, Daniel Chevallier, Matthieu Durand

**Affiliations:** ^1^ Urology, Andrology, Renal Transplant Unit, Hôpital Pasteur 2 CHU de Nice Nice France; ^2^ Department of Urology and Organ Transplantation University of Foggia Foggia Italy; ^3^ Department of Urology and Renal Transplantation, CHU Rangueil Toulouse University Hospital Toulouse France; ^4^ Department of Renal Transplantation, Hôpital Pasteur 2 Nice Sophia‐Antipolis University Nice France

**Keywords:** CD complications, delayed graft function, graft, kidney, night‐time, transplantation

## Abstract

**Introduction:**

Various surgical centers tend to postpone a kidney transplantation (KT) to the following morning than to operate at night‐time.

The objective of our study was to assess whether there was any difference between daytime and night‐time renal transplantation in our institution.

**Method:**

This study is a retrospective monocentric study including all the KTs that were performed between 2012 and 2013 by transplant expert surgeons in our institution. Clavien‐Dindo (CD) complications were classified according to 7 variables going from 1 to 5. Time before postgraft diuresis and delayed graft function (DGF) were also analyzed. Two groups of patients were formed according to threshold value of incision time (6.30 p.m.). Data comparison were performed using the Kruskal–Wallis nonparametric test.

**Results:**

A total of 179 patients were included. Median follow‐up was 24 months. Cold ischemia time was longer in the night‐time transplantation (1082 vs. 807 min, *p* < .001), but rewarming time was shorter (47.24 vs. 52.15 min, *p* = .628). No statistically significant differences were observed between the two groups using the Kruskal–Wallis method for CD complications (Qobs: 0.076; *p* = .735). CD complications proportion was similar, with a majority of grade II complications (72.7% daytime group vs. 75.4% night‐time group (*p* = .735). DGF (19 patients for daytime group vs. 13 patients for night‐time group, *p* = .359) and time before postgraft diuresis (4.65 days daytime group vs. 5.27 days night‐time group, *p* = .422) were similar between both groups. Multivariate analysis did not show significant predictors of CD complications Grade 3 and more.

**Conclusion:**

Night‐time renal transplantation did not induce more postoperative CD complications than diurnal procedures in our cohort, challenging the false preconceptions that allow surgical teams to delay this surgery.

## Graphical abstract

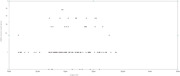

## 1 INTRODUCTION

Night‐time surgery has often been associated to a higher risk of postoperative complications, increasing both morbidity and mortality.^1^, ^2^ Night working after regular day work can cause fatigue and sleep deprivation, that could lead to an increase of malpractices.^3^ Reports from several countries also outlined the higher of anesthesia related‐incidents with those that carried out after‐hours.^4^

Kidney transplantation (KT) is considered as the Gold Standard treatment for end‐stage renal disease,^5^ but still hard to anticipate for surgical schedule. Limited supplies in grafts and increasing demand leads to patients not receiving a transplant in needed time, therefore increasing mortality rate on the waiting list. Multiorgan donor surgery is usually done during the evening or at night, since decisions on donors are mainly done during working day hours.^6^ Choice of allocation, transport of the recipient, and cross match, often takes 12–20 h,^7^ bringing most of the KT to office hours.

In 2019, 3641 renal grafts were transplanted in France (+4% increase compared to 2018) (https://www.agence-biomedecine.fr/IMG/pdf/cp_presentation-activite-greffes-annee-2019.pdf). KT is a challenging situation in surgical team organization, because of several features. First of all, this population is exposed to a higher risk for perioperative morbidity and mortality due to the severity of their disease.^8^ Second, since this surgery is an emergency procedure, the starting time of an intervention cannot be predict in advance, constrained by Cold Ischemia Time^9^ and availability of donor organs. A large number of surgical teams tend to delay surgery to the following morning, mainly due to the shortness of medical staff, and regulatory restrictions by law, forbidding physicians to work the following day after a night surgery, and therefore interfering with team's Operating Room (OR) schedules. This unnecessary prolongation of cold ischemia time (CIT) may have major influence on postoperative graft function, such as acute rejection, risk of delayed graft function (DGF), and even mortality.^9^, ^10^ To avoid this, surgeons should consider surgery at night in terms of renal transplantation with better understanding of possible complications due to night‐time KT.

The objective of our study was to assess whether there was any difference of postoperative complications in the 30 first days following KT, in patients that underwent either a night‐time transplantation (6.30 p.m. to 8 a.m.), nor a daytime KT (8 a.m. to 6.30 p.m.).

## 2 PATIENTS AND METHODS

### 2.1 Study cohort

We retrospectively reviewed data that were extracted from a prospective record collection, the French National Renal Transplantation database (DIVAT), of all recipients with a single KT from January 2012 to December 2013, in our University Hospital. DIVAT records prospectively and exhaustively the renal transplantation activity either from living or deceased donor, and is in accordance with the Helsinki Declaration of 1975. Patients included in DIVAT gave their informed consent before their inclusion in the database. Recipient's characteristics (demographic data, primary renal disease and follow‐up time after transplantation). Kidneys were either preserved in cold ice or preserved on pump, following French Transplantation guidelines regarding kidney preservation. Main information concerning each procedure was extracted: operating time, CIT and rewarming time (RT), Clavien‐Dindo (CD) complications, acute rejection, DGF and time before postgraft diuresis. CD complications were classified depending on 7 variables from 1 to 5.^11^ Peri and postoperative uses of immunosuppressive therapies were collected. Recipients received polyclonal antilymphocyte antibodies or anti IL2‐R for induction while maintenance immunosuppression consisted of calcineurin inhibitors, mycophenolate mofetil, or azathioprine and steroids.

### 2.2 Surgical technique

Surgery was done by several experienced surgeons, in our transplantation reference centre. Pararectal or Jalaguier‐Gibson incision was performed. Anastomoses were made end‐to‐side on external iliac vessels. Uretero‐vesical reimplantation was performed with the Campos Freire‐Lich‐Gregoire technique and a double J stent was left in place for 6 weeks.

### 2.3 Groups constitution

The cut‐off incision time was chosen according to the night shift time in the emergency ORs, both medical and paramedical teams. 6.30 p.m. was chosen as the threshold for separating daytime and night‐time surgery.

The primary study endpoint was the distribution of complications within each group. Secondary endpoints were the overall rate of complications, for each of the 7 CD complication variables, and time before postgraft diuresis.

### 2.4 CD complications

We reported all data regarding surgical and medical complications in the 30 first days, with or without revision, classified as CD complications^11^: number and dates of overall surgical revisions, ureteral complications (stenosis, fistulas), vascular complications (arterial and venous thrombosis and stenosis), haemorrhagic complications (haematomas, number of transfused unit of blood), lymphoceles, abscesses, eventrations, early graft explantation, use of drugs such as antiemetics, antipyretics, analgesics, diuretics, electrolytes, antibiotics, and use of dialysis.

### 2.5 Time before postgraft diuresis and DGF

Our institution evaluation of postgraft diuresis is based on the number of days when recipient's serum creatinine after renal transplantation matches with donor's kidney serum creatinine at time of procurement. DGF was defined as a need for dialysis in the first postoperative week.

### 2.6 Data collection and statistical analysis

Donor and recipient's preoperative data were extracted from DIVAT, while consultation, hospitalization, operative reports, biological and imaging results were extracted from the Clinicom software (Intersystems) used in our centre.

Statistical analysis was performed using Stata software. Statistical significance was defined as a *p* value <.05. Results are expressed as percentages for categorical variables, means ± *SD* for variables with a normal distribution and as medians for variables with a non‐normal distribution.

The difference in distribution of complications was assessed by the nonparametric Kruskal–Wallis test. Proportions were compared using *χ* ^2^ test, and mean time before diuresis, using student test. Multivariate analysis was done on CD complications grade 3 or more, and DGF.

We also represented the data by a plot of the CD complication depending on the time of incision, to visually evaluate an eventual tendency, and assess the robustness of our cut‐off time. Only two‐tailed tests were used, with first species risk of 5%.

## 3 RESULTS

A total of 179 patients who underwent KT during our inclusion period were retrospectively included for this study. Main characteristics are shown in Table 1, the two groups were comparable in terms of sex, kidney disease and comorbidities, but different in terms of donor's age (59.3 vs. 64.74, *p* = .024), recipient's age (55 vs. 61.26, *p* = .006) and Charlson score (4.4 vs 5.35, *p* = .005).

**Table 1:** Preoperative characteristics of the daytime and night‐time transplantation population

	Daytime transplantation (*n* = 122)	Night‐time transplantation (*n* = 57)	*p* value
Recipient age (mean [*SD*])	55 (14.65)	61.26 (12.98)	.006
Graft donor age (mean [*SD*])	59.35 (13.7)	64. 74 (13. 78)	.024
Sex			.654
Male	89 (73%)	39 (68.4%)	
Female	33 (27%)	18 (31.6%)	
Charlson score (mean [*SD*])	4.4 (2.0)	5.35 (2.01)	.005
Kidney disease			
Vascular	21 (17.2%)	13 (22.8%)	.494
Diabetes	14 (11.5%)	17 (29.8%)	.005
Glomerular	26 (21.3%)	4 (7%)	.030
Polycystic kidney	16 (13.1%)	6 (10.5%)	.805
Interstitial	11 (9%)	6 (10.5%)	.962
Undertermined	36 (29.5%)	11 (19.3%)	.206
Smoker, *n* (%)	108 (88.5%)	55 (96.5%)	.144
Comorbidities, *n* (%)			
Vascular	103 (84.4%)	51 (89. 5%)	.499
Cardiac	37 (30.3%)	11 (19. 3%)	.170
Endocrinian	65 (53.3%)	38 (66. 7%)	.127
Urologic	13 (10.7%)	2 (3. 5%)	.187
Hepato‐gastro‐enterologic	17 (13.9%)	10 (17. 5%)	.686

Ischemia times of the transplant are shown in Table 2. CIT was significantly higher in the night‐time group (1082.22 min vs. 807 for daytime group, *p* < .001). RT was not significantly different.

**Table 2:** Peroperative characteristics of the daytime and night‐time transplantation population

	Daytime transplantation (*n* = 122)	Night‐time transplantation (*n* = 57)	*p* value
Cold ischemia time, minutes (mean [*SD*])	807.04 (475.96)	1082 (224.17)	<.001
Warm ischemia time, minutes (mean [*SD*])	52.15 (74.14)	47.24 (16.4)	.628

**Figure 1:**
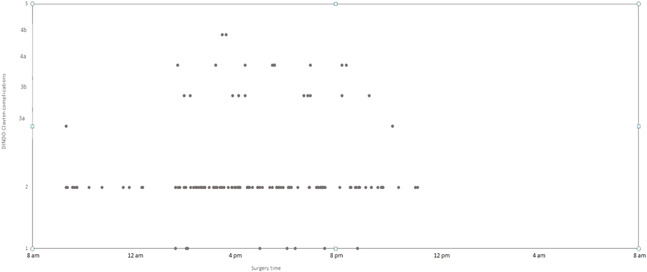
Distribution of Clavien‐Dindo complications according to surgery time using PROC PLOT procedure

**Figure 2:**
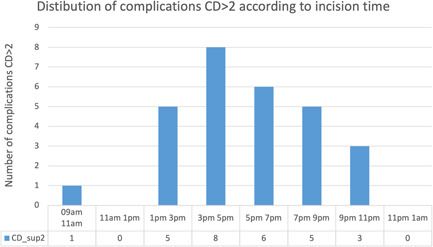
Distribution of Clavien‐Dindo complications >2 according to stratified incision time

**Figure 3:**
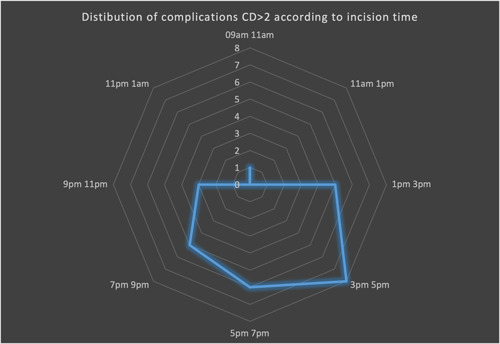
Distribution of Clavien‐Dindo complications >2 according to stratified incision time

Regarding the primary endpoint, we showed no significant differences in terms of postoperative CD complications between daytime and night‐time transplantation (Qobs = 0.076, *p* = .735). Table 3 and Figure 1 demonstrates the CD complications distribution between the two groups. The postoperative major complications (CD >2) were similar between daytime and night‐time group (18/122 [14.8%] vs. 10/57 [17.5%] in daytime and night‐time groups, respectively, *p* = .7). We then stratified for complications CD >2 depending on the time of incision, showing the highest complication rate for an incision time between 3 and 5 p.m. (Figures 2 and 3). There were no deaths reported in our cohort.

**Table 3:** Clavien‐Dindo complications repartition for daytime and night‐time transplantation

Clavien‐Dindo grade complications	Daytime transplantation	Night‐time transplantation	*p* value
I	15 (12.4%)	4 (7%)	.735
II	88 (72.7%)	43 (75.4%)	
IIIa	1 (0.8%)	1 (1.8%)	
IIIb	9 (7.4%)	6 (10.5%)	
IVa	6 (5%)	3 (5.3%)	
IVb	2 (1.7%)	0 (0%)	

**Figure 4:**
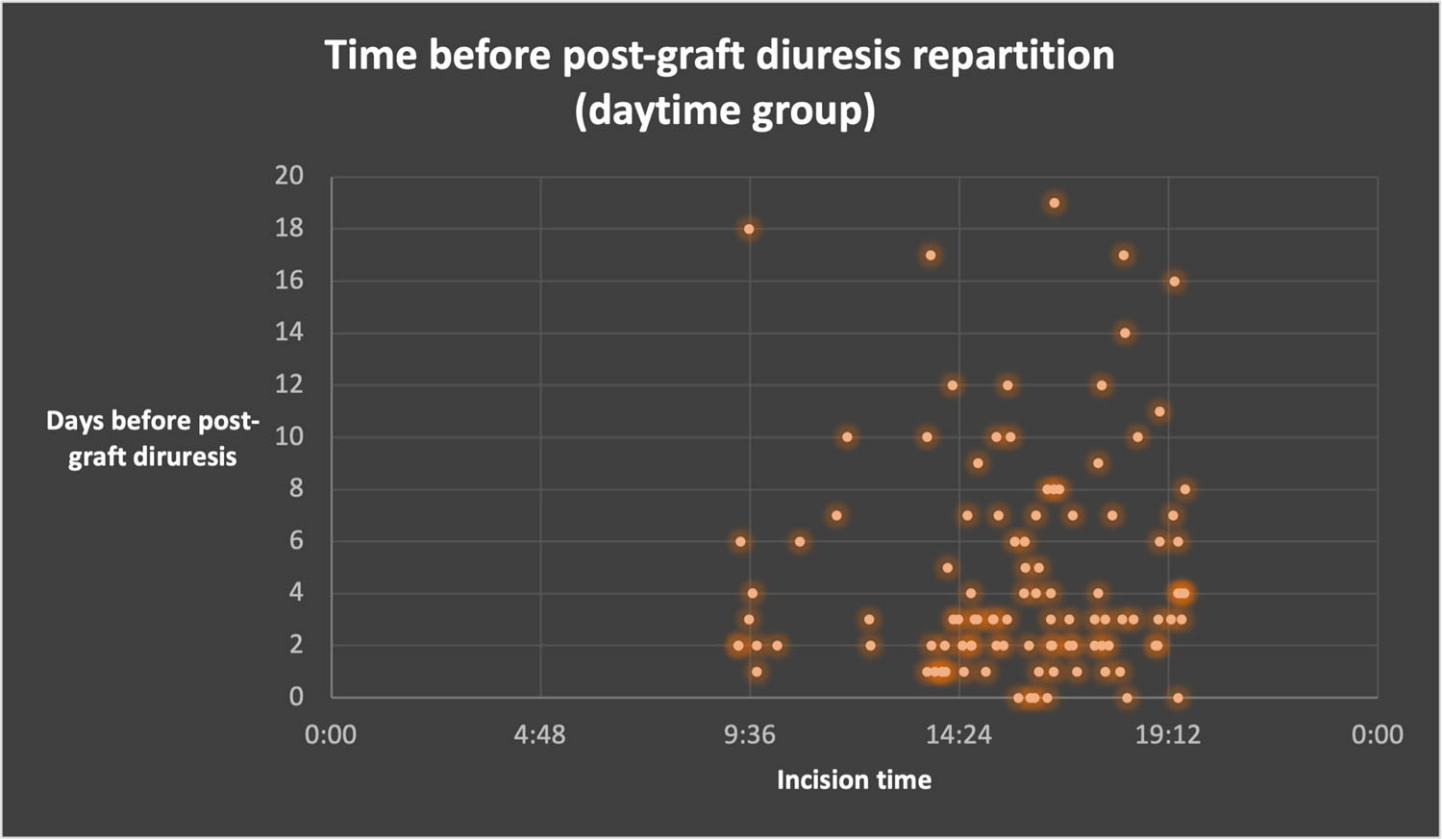
Time before postgraft diuresis dot plot repartition, in the daytime and night‐time group

**Figure 5:**
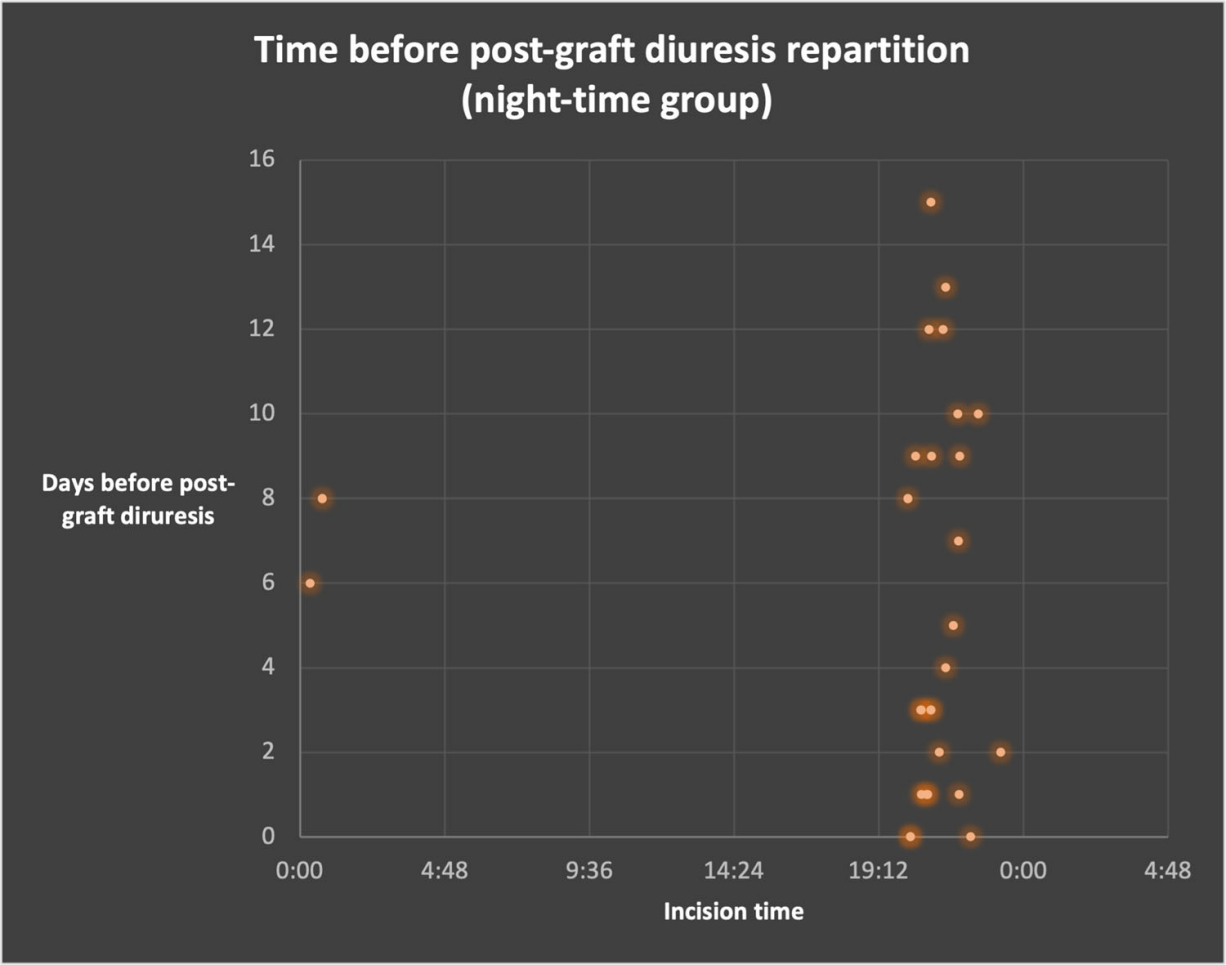
Time before postgraft diuresis dot plot repartition, in the daytime and night‐time group

Regarding secondary endpoints, DGF and time before postgraft diuresis were similar between both groups (*p* = .359 for DGF, *p* = .422 for time before postgraft diuresis, Table 4). There were no patients with a number of days before postgraft diuresis above 16 days for the night‐time group (Figures 4 and 5).

**Table 4:** Postoperative characteristics of the daytime and night‐time transplantation population

	Daytime transplantation	Night‐time transplantation	*p* value
Resistance index Day 1 (mean [*SD*])	0.72 (0.17)	0.82 (0.17)	.02
Resistance index Day 7 (mean [*SD*])	0.7 (0.11)	0.77 (0.13)	.021
Post‐graft diuresis (days, mean [*SD*])	4.65 (4.81)	5.27 (4.32)	.422
Acute graft rejection, *n* (%)	4 (3.7)	2 (4.5)	1.000
Number of postgrat dialysis, *n* (mean [*SD*])	0.62 (2.81)	0.53 (1.39)	.812
Delayed graft function, *n* (mean [*SD*])	19 (15.8)	13 (22.8)	.359

In our multivariate analysis, no variables were significant regarding CD complications >2 (Table 5). Regarding DGF, only age was at risk of a longer DGF (odds ratio: 1.05, 95% confidence interval [CI]: 1.005–1.106, *p* = .0407) (Table 6).

**Table 5:** Multivariate analysis on Clavien‐Dindo 3 and more complications predictors

	Odds ratio	*p* value
Night‐time transplantation	0.96 (0.37–2.37)	.92
Sex	0.98 (0.37–2.40)	.95
Recipient age	0.98 (0.94–1.01)	.20
Charlson score	1.25 (0.98–1.59)	.07
Cold ischemia time	1.02 (0.95–1.09)	.58
Warm ischemia time	1.00 (0.99–1.00)	.97

**Table 6:** Multivariate analysis on delayed graft functions predictors

	Odds ratio	*p* value
Night‐time transplantation	1.09 (0.45–2.56)	.85
Sex	1.59 (O.65–3.79)	.3
Recipient age	1.05 (1.01–1.11)	.04
Charlson score	0.96 (0.78–1.24)	.78
Cold ischemia time	1.06 (0.99–1.14)	.08
Warm ischemia time	1.01 (0.99–1.01)	.24

Regarding the description of CD complications, daytime group had 10 CD3 complications, with 1 urinary tract infection needing double J stenting, 2 lymphoceles needing interventional radiology for drainage, 5 redo surgeries for hematomas, 1 artery and 1 venous thrombosis. There were also 8 CD4 complications, with 1 acute respiratory failure needing high volume oxygen, 1 resuscitation after cardiac arrest, 4 acute coronary syndrome needing emergency stenting, 1 acute pulmonary oedema, and 1 Hemolytic Uremic Syndrome.

Night‐time group had 8 CD3 complications: 2 urinary tracts infections needing double J stenting, 1 redo surgery for hematoma, 1 lymphocele needing interventional radiology for drainage, 2 marsupializations for lymphocele, 1 redo surgery for renal vein thrombosis and 1 fat embolism syndrome. There was also 2 CD4 complications, with 1 renal vein thrombosis needing emergency explantation and 1 Atrial Fibrillation with the need for cardioversion.

## 4 DISCUSSION

Night‐time surgery has often been associated to a higher morbidity or mortality rate, previous studies confirming these hypotheses.^1^, ^2^ However, the primary outcome of our study confirmed the results of several other studies that contradicted the correlation between night‐time surgery and worse clinical outcomes, such as postoperative complications or graft failure. Kienzl‐Wagner et al.^12^ showed no differences in postoperative complications, and even less acute rejection (22.6% vs. 18.3% in night‐time graft recipients, *p* = .15) for night‐time KT. In a meta‐analysis regarding transplantations performed outside regular working hours, a 1.01 mortality Hazard ratio (95% CI: 0.98–1.04) was shown regarding “non‐regular” hour transplantations.^13^ Another interesting point in our study is that there was not any difference regarding CD complications, even though there was a significant difference in terms of donor, recipient age, and Charlson score, between the two groups (Table 1).

Regarding the distribution of CD complications, we did not find any significant differences between the two groups. There was more 3b CD complications in the night‐time group, however, these results were not significant. However, a larger population on this matter could provide a better stratification of the CD complications repartition. An interesting comparison would also be the peroperative complications, such as bleeding or vascular stenosis, between daytime and night‐time surgery. Interestingly, Brunschot et al.^14^ showed less pure technical graft failure in the night‐time renal transplantation (*n* = 4519), which contrast with Fechner's study,^15^ that showed more significant vascular stenosis (8.5% vs. 1.6%, *p* < .1, *n* = 260) in his night‐time population.

We also found a tendency to DGF for the night‐time transplant group on univariate analysis. On multivariate analysis, only patient's age had a real impact on DGF. DGF has a significant clinical and long‐term impact on the survival and functionality of the future graft.^16^ In other studies, various preoperative factors including donor age, high blood pressure, prolonged RT and pelvic atherosclerosis were found to promote ischemic injury and DGF, that we did not confirm in our study.^17^

Our two groups differed in terms of recipient's age, but in multivariate analysis, only age was at significant risk of DGF. These results complement Guo et al's study, where kidney donors over 60 had higher 1 or 2 CD complications, but no difference in 3 or 4 groups; however, older patients had a significantly lower eGFR at 1 month and 1 year.

In our study, 6.30 p.m. was chosen as a threshold for incision time to differentiate daytime and night‐time population. This threshold differs from most of the other studies comparing daytime and night‐time surgery, with a 8 p.m. incision time as a cut‐off. 6.30 p.m. is the switch time between both nurse and anaesthesiologist team in our center. That time is crucial, with a transfer of all the perioperative informations between teams, and can lead to transmission error. Giugale et al.^18^ showed an increase surgery time in the nurse handoff period, but no major CD complications associated during that time. Other studies reported higher risk of postoperative complications or even mortality when anaesthetic handover was done during cardiac surgery.^19^ This time is also the on‐duty time for surgeons in our institution, meaning the start of a night shift after a whole day of surgery or consultations, introducing fatigue and decrease in concentration. Night‐time surgery in our institution is also at risk of resource limitation, as there is fewer paramedical staff available than during the day, and OR nurses are not specialized in urology surgery or KT, creating an additional stress factor for the surgeon during the surgery. During daytime, a specialized urology paramedical team is dedicated to KTs (two experienced OR nurses in KT, and a specialized anaesthesiologist assistant). At night‐time, KT are done in normal emergency OR, with OR nurses that are capable of all general emergency surgeries but not specialized in KT, therefore not always aware of the different important moments of the surgery.

The success of a KT is mostly due to a short cold ischaemia time,^20^ and an 18 h CIT threshold has been associated to fewer postoperative complications.^21^ We found in our study a significant difference in terms of CIT, with a longer CIT within the night‐time group (Table 2). In our centre, deceased donor kidneys coming from other institutions tend to arrive in the middle of the night or early morning, and KT is postponed to the following day at the end of the programmed surgery schedule. This is caused firstly by the lack of dedicated operation rooms for transplantation, and secondly waiting on the recipient to arrive at the institution and get prepared for the transplantation. In contrary, a multiorgan retrieval from deceased donor in our institution is usually performed during the night, and allows both surgeons and transplantation doctors a longer preoperative time to organise a KT for the following day. Delayed surgery to avoid night‐time induces a risk for the graft function,^10^ and there is actually no medical reason to further delay the surgery. However, the significant difference between daytime and night‐time group regarding CIT did not show consequences regarding CD complications, DGF, or time before postgraft diuresis.

However, it is common sense that excessive duty hours and extended work shifts (24 h or more) inevitably leads to sleep deprivation, which is one of the risk factors of burn out.^22^ Another factor of extended cold ischemia time is the “competition” between KT surgeries and other type of elective or emergency surgeries done in the common emergency ORs, due to either lack of paramedical staff or OR availability. To overcome this issue, a parallel and independent organization with a dedicated room and team would be able to reduce this ischemia time without impacting the overall institution surgical planning; however, this organization can only be done in high‐volume transplantation centres.

We recognise several limitations in our study. First, we looked at postoperative CD complications, and DGF, not taking into account the clinical follow‐up regarding kidney graft function or mortality. It would be interesting to monitor this population to evaluate long‐term effects of night‐time transplant surgery, regarding renal function and tardive complications. There are no studies comparing day and night transplants regarding recovery of renal function or hypertension occurrence, but it has already been showed that night‐time renal transplantation had an impact on transplant's lifespan.^15^ Another interesting subanalysis would be to look at that different time slots during night‐time surgery. Is it conceivable that late evening surgery would not have the same impact on a surgeon's fatigue and concentration as a procedure done in the middle of the night or early morning.^23^

Our short period of inclusion and absence of contemporary data is also a limitation factor, due to a change in the local KT surgical organization, cancelling all night‐time transplantations in our centre after 2013 because of organizational restrictions. Thirdly, our small population sample and our retrospective analysis are understandably a limit to conclude in night‐time KT being as safe as daytime transplantation regarding postoperative complications.

Finally, a comparison between weekday and weekend transplants could add to the analysis regarding surgical planning, as various studies have shown both more postoperative complications,^24^ an increase in the odds of kidney discards during weekend^25^ and increased mortality regarding weekend surgeries^26^

## 5 CONCLUSION

Our study confirms the absence of difference in the CD postoperative complications between night‐time and daytime KT in our cohort, challenging the false preconceptions that allow surgical teams to delay this surgery, furthermore increasing the risk of kidney graft failure or acute rejection as the cold ischemia time increases. Above all, the main goal of reducing the CIT should be considered. Based on evidence‐based medicine, night‐time KT should no longer be avoided for fear of complication. To do so, organizational boundaries of night‐time KT must be addressed by focusing on dedicated surgical teams and easy access to OR.

## CONFLICT OF INTERESTS

The authors declare that there are no conflict of interests.

## ETHICS STATEMENT

All human studies have been reviewed by the appropriate ethics committee and have therefore been performed in accordance with the ethical standards laid down in an appropriate version of the 2000 Declaration of Helsinki as well as the Declaration of Istanbul 2008.

## Data Availability

The data that support the findings of this study are available from the corresponding author upon reasonable request.
